# Ameloblastic Fibrodentinoma Presenting as a False Gingival Enlargement in the Maxillary Anterior Region

**DOI:** 10.1155/2015/812087

**Published:** 2015-01-29

**Authors:** Shiny Joseph, Lakshmi Priya, Dinesh Gopal, Mary Devachen, Ajay Narayan, Muhammed Afnan

**Affiliations:** ^1^Department of Periodontics, Al-Azhar Dental College, Thodupuzha, Kerala, India; ^2^Department of Oral Pathology and Microbiology, Al-Azhar Dental College, Thodupuzha, Kerala, India; ^3^Department of Oral and Maxillofacial Surgery, Al-Azhar Dental College, Thodupuzha, Kerala, India; ^4^Department of Oral Medicine and Radiology, Al-Azhar Dental College, Thodupuzha, Kerala, India; ^5^Department of Pedodontics, Al-Azhar Dental College, Thodupuzha, Kerala, India

## Abstract

Ameloblastic fibrodentinoma is a rare benign mixed odontogenic neoplasm usually occurring in the first two decades of life. It is more common in males and the most common site of occurrence is in the mandibular premolar molar area. This report presents a case of ameloblastic fibrodentinoma in a 12-year-old boy in the maxillary anterior region, a less common site for the occurrence of ameloblastic fibrodentinoma. A 12-year-old boy presented with a midline diastema in 11 and 21 region and a swelling in the palatal aspect of 11 and 12. Intraoral periapical radiograph showed the presence of rarefaction of bone on the mesial aspect of the cervical and middle third of the root of 11. Excision biopsy was done. The specimen was processed and stained with hematoxylin and eosin. Microscopic examination showed islands, chords and strands of odontogenic epithelium in a primitive ectomesenchyme resembling dental papilla. The odontogenic epithelium exhibited peripheral ameloblast-like and central stellate reticulum-like cells. The presence of dentinoid material was seen adjacent to the odontogenic epithelium in some foci. The lesion was diagnosed as ameloblastic fibrodentinoma.

## 1. Introduction

Ameloblastic fibrodentinoma also called dentinoma, arising from the odontogenic epithelium and ectomesenchyme, is a rare benign mixed odontogenic tumor with an occurrence of less than 1% of the odontogenic tumors [1]. The mixed odontogenic tumors which also include ameloblastic fibroma and ameloblastic fibroodontoma are a group of tumors of odontogenic epithelium and mesenchyme with or without dental hard tissue formation.

The majority of these hamartomatous lesions were observed during the first two decades of life, but it has been reported in older individuals as well. Ameloblastic fibrodentinoma is a slow growing asymptomatic lesion with both central and peripheral counterparts. There is a distinct male preponderance with a male: female ratio of 3 : 1 and it is usually seen in the mandibular premolar molar area [2]. Sometimes ameloblastic fibrodentinoma is associated with an unerupted tooth, in which case the tumor is associated with the crown [3, 4]. The tumors related to deciduous teeth usually arise in the incisor area while those related to permanent teeth develop in the molar area [5]. Although the lesion has a tendency to recur, enucleation of the lesion and curettage will suffice. The chance of ameloblastic fibrodentinoma to progress to its malignant counterpart ameloblastic fibrosarcoma is rare [6].

## 2. Case Description

**Figure 1:**
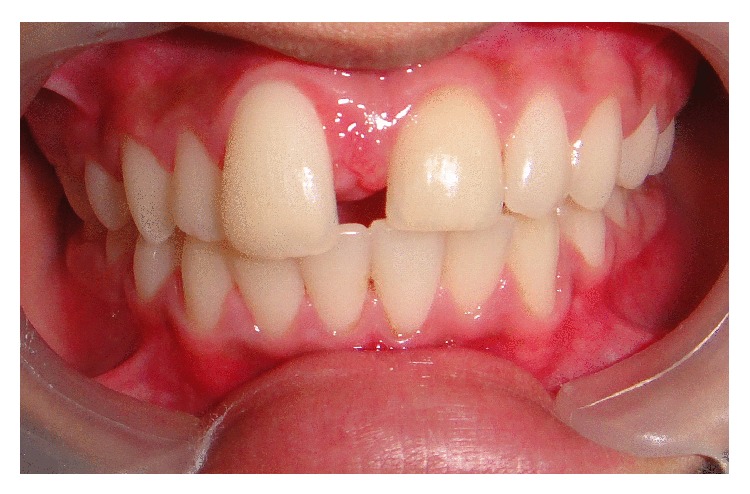
Spacing between 11 and 21 with extrusion and slight labial displacement of 11.

**Figure 2:**
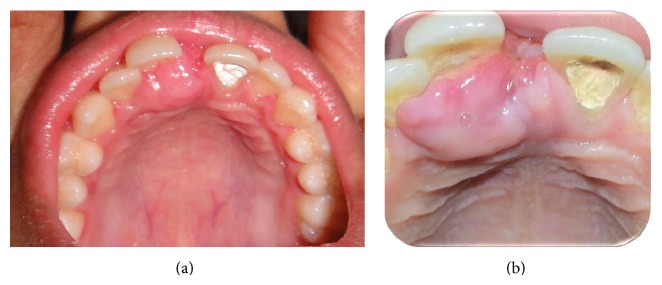
Well-defined enlargement on the palatal aspect of 11, involving the marginal and attached gingiva, and extending into the midline area.

**Figure 3:**
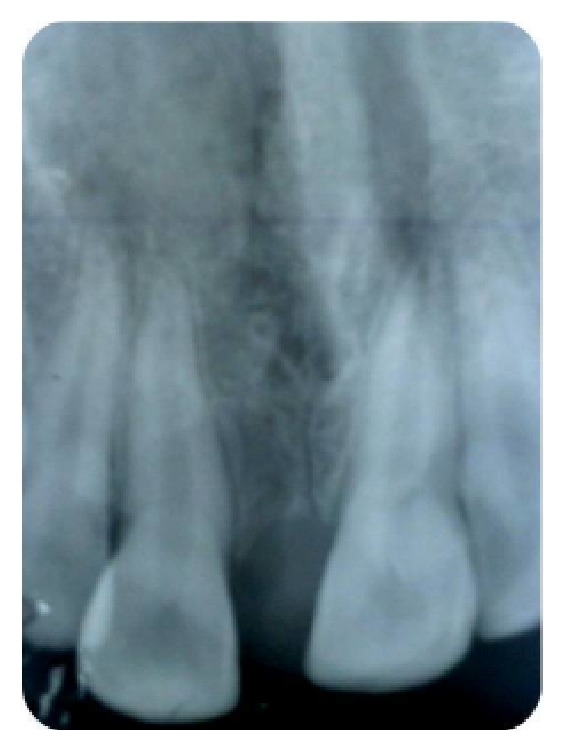
Rarefaction of bone on the mesial aspect of the cervical and middle third of the root of 11.

A 12-year-old boy came to the Department of Oral Medicine and Radiology with a chief complaint of a growth of the gingiva on the palatal aspect of 11. The growth appeared along with the eruption of the upper front teeth. At the time of eruption, there was midline diastema in 11 and 21 region with a nodule of tissue in between, which the patient pinched out 1 year back (Figures 1, 2(a), and 2(b)). He gave a history of trauma to the anterior teeth 8 months back. On intraoral examination, full complement of permanent teeth except the third molars was present. A midline diastema of 0.5 cm was present between 11 and 21 with slight labial displacement and extrusion of 11. There was a well-defined swelling of 2 × 2 cm size on the palatal aspect of 11, 12 involving the marginal and attached gingiva that extended into the midline area. It was pale pink in color with an intact epithelium and a nodular surface. It was nontender, soft in consistency, nonfluctuant, compressible, and nonindurated. Radiographic examination showed spacing between 11 and 21 with the alveolar crest at the cementoenamel junction, rarefaction of bone on the mesial aspect of the cervical and middle third of the root of 11, and a diffuse periapical radiolucency in relation to 21. There was no gross alteration in the trabecular pattern (Figure 3).

**Figure 4:**
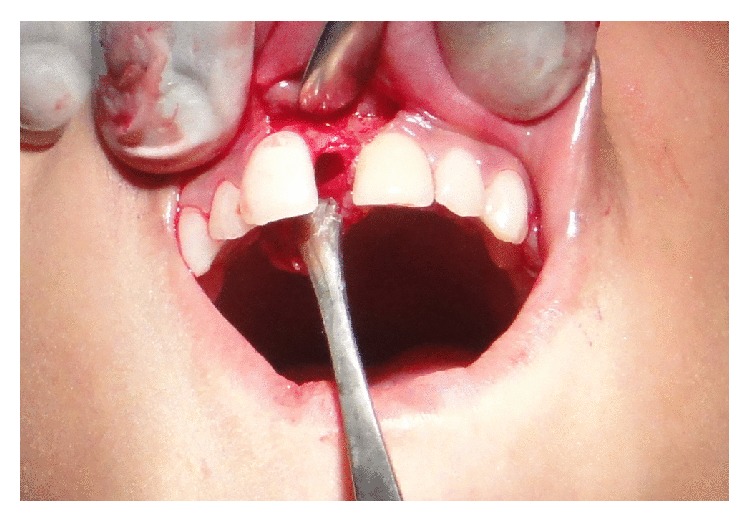
Cavitary space in the nasopalatine region after the granulomatous tissue was curetted out.

Excisional biopsy of the lesion was done. The gross specimen was a granulomatous tissue in the nasopalatine region in a cavitary space with regular margins and smooth floor (Figure 4). The tissue was curetted out from the bony cavity and after curettage, the bony margins were observed to be smooth. The enucleated granulomatous tissue was soft in consistency and about 0.5 × 1 cm in size.

**Figure 5:**
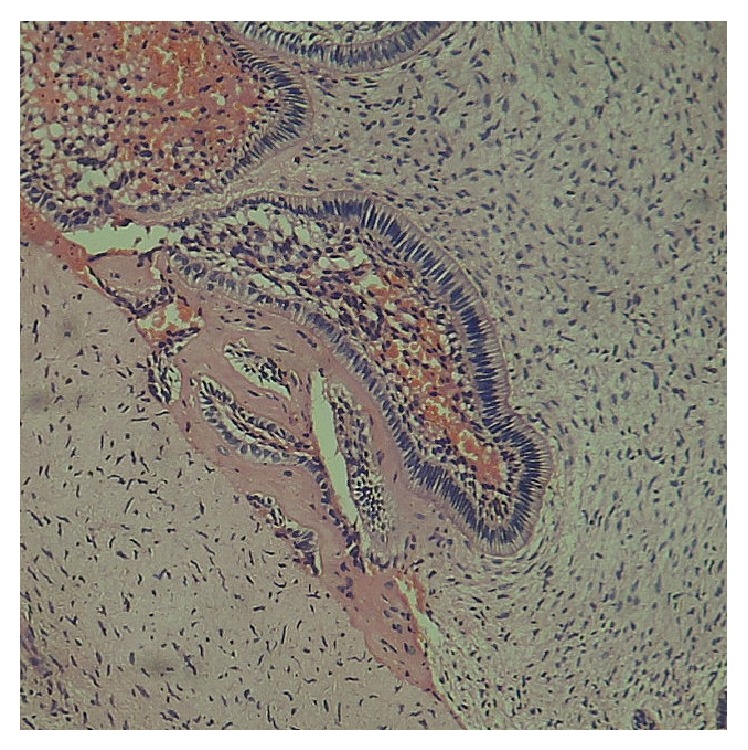
This figure shows an enamel organ type of odontogenic epithelium in association with an odontogenic ectomesenchyme. The hyalinised area containing entrapped cells is considered as dentinoid tissue due to its proximity to the odontogenic epithelium.

On microscopic examination of the specimen, islands, chords, and strands of odontogenic epithelium in a primitive ectomesenchyme resembling dental papilla were observed. The odontogenic epithelium exhibited peripheral ameloblast-like and central stellate reticulum-like cells. Presence of dentinoid material was seen adjacent to the odontogenic epithelium in some foci (Figure 5). A final diagnosis of ameloblastic fibrodentinoma was made.

## 3. Discussion

Ameloblastic fibrodentinoma is a slow growing, asymptomatic lesion, generally occurring in the first two decades of life, with the posterior region of the mandible being the most common site of occurrence [2]. It is sometimes associated with the crown of an unerupted tooth, and when the lesion occurs in relation to a deciduous tooth, the common site of presentation is the anterior maxilla [3, 4]. In our case also the lesion was slow growing and asymptomatic with the age of the patient coinciding with the normal age group in which the lesion occurs. However, this case presented a rarity in that it occurred in the anterior maxillary region, a less common site for the occurrence of ameloblastic fibrodentinoma. Full complement of teeth proportionate to the age of the patient was present. Radiographically supernumerary teeth were not identified and the lesion was noticed only after eruption of the permanent maxillary central incisors. This negates the possibility of the lesion to have developed in relation to the crown of a permanent tooth or in relation to a deciduous tooth.

Ameloblastic fibrodentinoma is an odontogenic tumor with or without formation of dental hard tissues. Radiographically, it presents as a well-defined radiolucency with varying degrees of radioopacity. Our radiograph also showed the presence of a well-defined radiolucency in relation to the mesial aspect of the cervical and middle third of the root of 11 surrounded by a corticated border without any detectable radioopacities within the radiolucency.

Histologically ameloblastic fibrodentinoma is composed of proliferating epithelium embedded in a cellular ectomesenchymal tissue that resembles dental papilla, and the proliferating epithelium exhibits varying degrees of inductive changes on the mesenchyme, leading to the formation of varying amounts of dentin [7]. If there is formation of both dentin and enamel, it is referred to as ameloblastic fibroodontoma [8]. In this case, microscopic examination of the collected specimen showed presence of dentinoid material adjacent to the odontogenic epithelium. Multiple sections were examined but none of them showed presence of enamel. Hence the lesion was diagnosed as ameloblastic fibrodentinoma.

Confusion exists on the nature and interrelationship of the mixed odontogenic tumors. Some authors are of the view that all the three mixed odontogenic tumors are interrelated and that ameloblastic fibrodentinoma is an intermediate stage between ameloblastic fibroma and ameloblastic fibroodontoma depending on the degree of histodifferentiation [9, 10]. They regard the lesions as chronological stages in a continuum in the following order: ameloblastic fibroma, ameloblastic fibrodentinoma, ameloblastic fibroodontoma, and odontoma [11]. However, in the 33 mixed odontogenic tumors analysed by Slootweg, the mean age of the patients diagnosed with ameloblastic fibroma was higher as compared to the patients diagnosed with ameloblastic fibroodontoma [12]. This refutes the assumption that the mixed odontogenic tumors are interrelated but that ameloblastic fibrodentinoma is an independent lesion and not one that is differentiated from ameloblastic fibroma. Ameloblastic fibrodentinoma also has a different biological behaviour as compared to ameloblastic fibroma [11].

Based on the stage of development, ameloblastic fibrodentinoma can be histologically divided into immature and mature type. The malignant counterpart of ameloblastic fibrodentinoma is ameloblastic fibrodentinosarcoma. The mechanism of malignant transformation of ameloblastic fibrodentinoma is unknown. Multiple surgical procedures of recurrent lesions have been suggested as an important factor for the malignant transformation. Metastasis of the malignant counterpart is also uncommon [11].

## 4. Conclusion

This case presented a rarity in that the lesion presented clinically as a case of gingival enlargement in the palatal aspect of the maxillary anterior region. Presence of a well-defined radiolucency on radiographic examination suggested that it was a case of false gingival enlargement, and the histopathologic examination confirmed the case as ameloblastic fibrodentinoma. Hence, this case is unique because of the fact that an intraosseous ameloblastic fibrodentinoma presented as a false gingival enlargement in the anterior maxillary region, a less common site for the occurrence of ameloblastic fibrodentinoma. This case highlights the importance of thorough evaluation of any gingival enlargement to rule out the possibility of an underlying neoplastic process.

## References

[1] Taylor A. M. (2008). New findings and controversies in odontogenic tumors. *Medicina Oral, Patologia Oral y Cirugia Bucal*.

[2] Ghom A., Mhaske S. (2009). *Textbook of Oral Pathology*.

[3] Philipsen H. P., Reichart P. A., Prætorius F. (1997). Mixed odontogenic tumours and odontomas. Considerations on interrelationship. Review of the literature and presentation of 134 new cases of odontomas. *European Journal of Cancer Part B: Oral Oncology*.

[4] Chen H.-S., Wang W.-C., Lin Y.-J., Chen Y.-K., Lin L.-M. (2006). Gingival ameloblastic fibro-dentinoma—report of a case in a child. *International Journal of Pediatric Otorhinolaryngology*.

[5] Akal U., Gunhan O., Guler M. (1997). Ameloblastic fibrodentinoma: report of two cases. *International Journal of Oral and Maxillofacial Surgery*.

[6] Barnes L., Eveson J. W., Reichart P., Sidransky D. (2005). *World Health Organization Classification of Tumors. Pathology and Genetics Head and NeckTumors*.

[7] Neville B. W., Dam D. D., Allen C. M., Bouquot J. E. (2008). *Textbook of Oral and Maxillofacial Pathology*.

[8] Cavalcante A.-S. R., Anbinder A.-L., Costa N.-C. S., Lima J.-R. S., Carvalho Y.-R. (2009). Ameloblastic fibro-odontoma: a case report. *Medicina Oral, Patología Oral y Cirugía Bucal*.

[9] Kramer I. R., Pindborg J. J., Shear M. (1992). *Histological Typing of Odontogenic Tumors*.

[10] Gardner D. G. (1984). The mixed odontogenic tumors. *Oral Surgery Oral Medicine and Oral Pathology*.

[11] Takeda Y. (1999). Ameloblastic fibroma and related lesions: current pathologic concept. *Oral Oncology*.

[12] Slootweg P. J. (1981). An analysis of the interrelationship of the mixed odontogenic tumors—amelobastic fibroma, ameloblastic fibro-odontoma, and the odontomas. *Oral Surgery Oral Medicine and Oral Pathology*.

